# DNA Vaccines: Their Formulations, Engineering and Delivery

**DOI:** 10.3390/vaccines12010071

**Published:** 2024-01-11

**Authors:** Michael Kozak, Jiafen Hu

**Affiliations:** 1 The Jake Gittlen Laboratories for Cancer Research, College of Medicine, Pennsylvania State University, Hershey, PA 17033, USA; 2 The Department of Pathology and Laboratory Medicine, College of Medicine, Pennsylvania State University, Hershey, PA 17033, USA

**Keywords:** DNA vaccine, vector, adjuvant, delivery, TH1, TH2, immune responses, innate immunity, adaptive immunity, integration, tolerance, inflammatory, gene therapy

## Abstract

The concept of DNA vaccination was introduced in the early 1990s. Since then, advancements in the augmentation of the immunogenicity of DNA vaccines have brought this technology to the market, especially in veterinary medicine, to prevent many diseases. Along with the successful COVID mRNA vaccines, the first DNA vaccine for human use, the Indian ZyCovD vaccine against SARS-CoV-2, was approved in 2021. In the current review, we first give an overview of the DNA vaccine focusing on the science, including adjuvants and delivery methods. We then cover some of the emerging science in the field of DNA vaccines, notably efforts to optimize delivery systems, better engineer delivery apparatuses, identify optimal delivery sites, personalize cancer immunotherapy through DNA vaccination, enhance adjuvant science through gene adjuvants, enhance off-target and heritable immunity through epigenetic modification, and predict epitopes with bioinformatic approaches. We also discuss the major limitations of DNA vaccines and we aim to address many theoretical concerns.

## 1. Introduction

The advent of cell culture in the early 1900s [1], the development of the electron microscope in the early 1930s [2,3], the discovery of DNA structure and the central dogma in the 1950s [4] and sequencing technology in the 1970s [5] have contributed significantly to the development of vaccines for numerous previously morbid and mortal pathogens [6,7,8]. 

The most salient vaccine technology of the 21st century is gene vaccination. Gene vaccines work by harnessing advances in biochemistry, molecular biology, genetics and chemistry to deliver selected portions of the deoxyribonucleic acid (DNA), or messenger ribonucleic acid (mRNA), of the pathogen of interest. In theory, the engineering can eliminate any pathogenicity, thus reducing the morbidity of vaccination and obviating any mortality of vaccination owing to inoculum pathogenesis [9,10]. 

DNA vaccines, first developed in the 1990s [11], are scalable, stable, easily manipulable and amenable to stockpiling [12]. They produce comprehensively robust adaptive immune responses comparable to those seen in attenuated pathogen vaccines with the safety profile of a subunit vaccine and can so can be given to immunocompromised persons [13]. The earliest gene vaccines consisted only of DNA as plasmids, which were taken up by cells via different delivery methods, transported to the nucleus, transcribed to mRNA and then translated into proteins, which are processed to antigens that stimulate immune responses [11,12]. 

The mRNA vaccine is technically different, because mRNA is not packaged into a plasmid; however, the mRNA vaccine offers significant advantages over the DNA vaccine, the two most important advantages being that the antigen to be presented is post-RNA-processing and integration into the host genome is substantially less likely [14,15].

While gene vaccination is not a household term, ‘mRNA vaccine’ certainly is owing to the incredible successes of two mRNA vaccines during the SARS-CoV-2 global pandemic [16,17]. DNA and mRNA vaccines are both gene vaccines, and thus fundamentally converge at the point of immunogenic protein translation within the cell; however, DNA and mRNA vaccines are not entirely alike—a DNA vaccine does not simply become an mRNA vaccine when the nucleic acid sequence enters a cell and undergoes transcription, because the processing of DNA to RNA in vivo includes some degree of variability at the local (cell-specific) level [18], and RNA translation, likewise, is subject to local factors [19]. 

DNA vaccines work by delivering antigens in their genetic form, as DNA plasmids, to cells. Both DNA and mRNA vaccines need to cross the cell membrane, and, as hydrophilic, polar and charged molecules, they do not freely cross the cellular membrane. DNA must additionally cross the nuclear envelope to the nucleus to be transcribed to mRNA before the antigenic protein can be translated in order to stimulate an immune response. mRNA vaccines are post-processing, do not need to cross the nuclear envelope and can be immediately translated to the antigenic protein once in the cytoplasm [20]. 

DNA vaccines were first put forth in the early 1990s when it was discovered that intramuscular plasmid DNA injection could induce protein expression, and importantly, it was concurrently shown that no special delivery system was necessary for most cells to take up DNA and route it to the nucleus [21]. Shortly after, this idea was adapted to the delivery of antigens of interest [22], and it has since been shown that naked DNA delivered to the cell results in relatively low and inconsistent gene expression [23,24], so newer formulations were adapted and developed to increase expression. Technological advances have made the viral vector one of the platforms of choice for DNA delivery by placing the gene of interest within the genome of a modified virus [25]. The virus is then taken up by cells, in much the same way a viral infection would occur, after which the replicative property of the virus is harnessed for the expression of the delivered gene sequence [26].

It would be more than thirty years from discovery until the first DNA vaccine was approved for use in humans: the Indian ZyCovD [27,28,29], which is a vaccine against SARS-CoV-2 [27]. The first DNA vaccine to make it to a phase I clinical trial was an HIV-1 vaccine in the late 1990s [30]. While the HIV vaccine never made it to market, the proof of concept was satisfied and many of the safety concerns about the technology were tempered by the trial [31]. One of the principle hurdles to effective HIV vaccination is the rapid rate of mutation of HIV, a retrovirus with a famously error-prone reverse transcriptase and short generation time [32,33,34,35]. Mutating pathogens are a moving target for vaccination, and this is one of the principle reasons for the success of the COVID-19 vaccines: the spike protein is highly conserved [36]. As of today, hundreds of DNA vaccines are in active clinical trials, or have completed clinical trials, for conditions including: influenza, zika, breast cancer, HIV, prostate cancer, malaria, HSV, melanoma, hepatitis and others [37]. 

Had it not been for the pandemic, a different DNA vaccine would likely have made it to market first. Although, which vaccine this might have been is difficult to answer; several technical improvements have contributed to a renewed interest and expanded promise. These include more effective delivery approaches, adjuvant advancements, promoter and gene optimization strategies and improved nucleic acid design [30]. DNA vaccines are in wide use in veterinary medicine and many of the technological and engineering advances in trial today have come from veterinary research [12,38]. Human applications of the technology have lagged, largely due to sub-optimal immunogenicity when compared to conventional vaccine approaches [39]. Many technological advances have brought DNA vaccination forward, and more is yet to come.

DNA vaccines in clinical trials, and through clinical trials, are summarized in other reviews [40,41,42,43,44,45,46]. This review will focus specifically on DNA vaccine technology, including its development, applications and emerging technologies. However, many of the technologies being developed can also be readily applied to mRNA vaccines.

**Figure 1:**
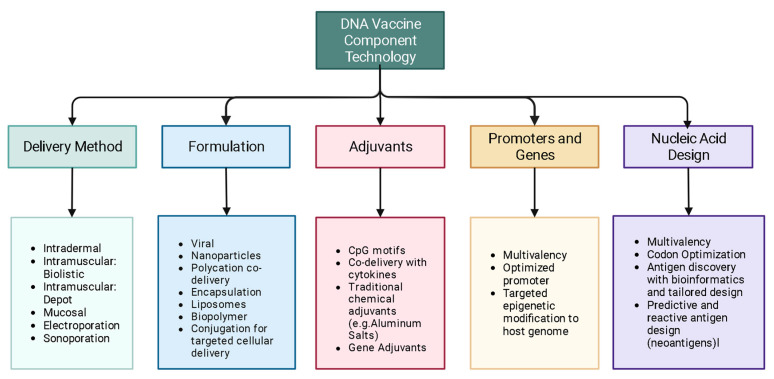
Some of the components of DNA vaccine technology. Many factors including delivery route, vector design, adjuvant choice and promotor/gene design all play important roles in the outcome of DNA vaccinations. Combinations of these different components make the DNA vaccine one of the most versatile yet challenging vaccine formats. Created with BioRender.com.

Some of the emerging science in the field of DNA vaccines, covered in this review, aims to: optimize delivery systems, better engineer delivery apparatuses, improve delivery techniques, personalize cancer immunotherapy through DNA vaccination, enhance adjuvant science through gene adjuvants, enhance off-target and heritable immunity through epigenetic modification, as well as to model and predict epitopes with bioinformatic approaches (Figure 1).

## 2. Mechanism

**Figure 2:**
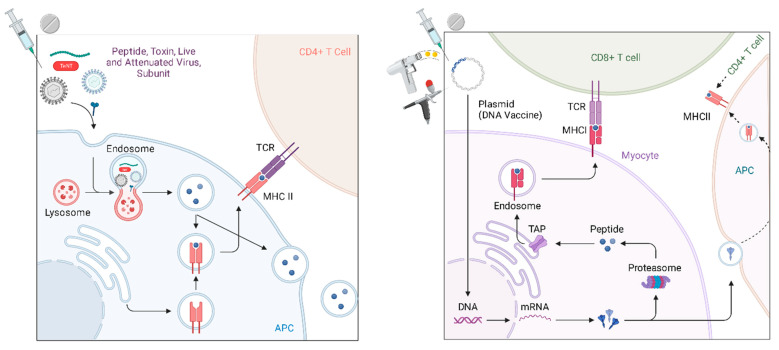
Conventional vaccine mechanism and DNA vaccine mechanism. Conventional vaccines (**on the left**) including peptide, subunit, live and attenuated viruses and toxins require endocytosis and intracellular processing of the pathogen. Because the pathogen is exogenous to the presenting cell, it is processed through the MHC II pathway, which preferentially engages CD4+ cells. DNA vaccine (**on the right**) can be endocytosed or can be engineered to passively cross the phospholipid membrane. The nucleic acid then locates to the nucleus and transcription and translation occur as if the DNA were native, which leads to presentation of the peptide through the MHC I pathway, preferentially activating CD8+ cells, additionally, the same peptide is exocytosed and then taken up by nearby cells, which then present the peptide via the MHC II pathway. Adapted from “COVID-19 DNA-Based Vaccine”, by BioRender.com (2023). Retrieved from https://app.biorender.com/biorender-templates accessed on 10 December 2023

Like all vaccines, the mechanism by which DNA vaccines generate immunogenicity is by activating the adaptive immune response. When a plasmid is delivered near to a cell and then taken up (either passively or by facilitation), the plasmid DNA can then be recognized and expressed by the native cellular machinery to generate the target antigen within the cell that has taken it up. From there, antigens (usually varying lengths of peptides) are presented on the cell surface for interaction with the immune cells by one of two pathways, either the major histocompatibility complex (MHC) class I (MHC I) or class II (MHC II) pathways (Figure 2). MHCI, which is present in all nucleated cells [47], is most frequently thought to be the presentation mechanism for endogenous antigens (most commonly peptides), while MHCII is thought to be the classical pathway for the expression of exogenous antigens, such as bacteria, fungi, protozoa and free viruses that the cell has endocytosed [48,49,50]. Because plasmids are taken up by the cell and the antigen to be presented is then generated intracellularly through the transcription and translation of the delivered DNA, the most common mechanism of the antigen presentation in DNA vaccination is MHC Class I [51,52]. 

Furthermore, MHC Class I presentation is associated with the activation of CD8 T lymphocytes; CD8 serves as an adhesion molecule for MHC I [52,53,54]. In sequence, DNA vaccines generate intracellular peptides, which are, in this pathway, endogenous proteins, and are most commonly presented on MHCI and activate CD8 T cells. Concurrently, the translated proteins are also exocytosed and subsequently taken up by other antigen presenting cells, which drain to regional lymph nodes [55]. These include macrophages, dendritic cells and other monocytes. These antigen presenting cells (APCs) endocytose and/or phagocytose materials from the extracellular environment and then process and present by MHCII, which preferentially activates CD4 T cells. Two types of CD4 T helper cells (Th1 and Th2) can further activate CD8 T cells or promote B cell differentiation to plasma cells to generate an humoral immune response, respectively [55]. The relative balance of both cytotoxic and humoral immunity [56] sets gene vaccines apart from other vaccination technologies, that have historically relied more heavily on humoral responses and have generated attenuated cytotoxic effects by antigen cross-presentation on MHCI [57] without necessarily ensuring a robust cell-mediated response [52,58]. Additionally, plasmid DNA has historically been derived from bacteria. As DNA, and because of its fundamental design, plasmids can have a number of CpG motifs. A CpG motif is a known adjuvant that activates TLR9-bearing B cells and plasmacytoid dendritic cells to promote strong Th1-type immune responses and these CpG motifs can be optimally engineered into DNA vaccines [59,60,61]. Likewise, viral vectors are chosen for their intrinsic immunostimulatory effects and for their facility in transporting their genomic cargo into cells.

The immune responses generated by a vaccine are an essential consideration. The specific T cell and B cell activation is especially crucial when determining a vaccine’s effectiveness. T cells have T-cell receptors (TCRs) that recognize processed antigen fragments presented on MHC by other cells [62], either cells that have endocytosed the antigen (bacteria) or cells that are producing the antigen (tumors or virally infected cells). These T cells need to differentiate between self and foreign peptides, and are only activated upon MHC presentation of foreign epitopes [63]. Regarding vaccination, T cells respond to a cell that is infected to primarily eliminate infected cells. So, T cells are primarily reactionary in the scope of a pathogenic invasion. On the other hand, B cells have receptors, specific to an epitope, which can bind to extracellular antigens. And, the B cell receptor has the same specificity as the antibodies that the terminally differentiated plasma cell secretes [62]. In conventional peptide, subunit, or inactivated pathogen vaccinology, antibody titers have been the measure of efficacy and a surrogate for preventative protection [64].

Plasma cells secrete neutralizing antibodies against the pathogen of interest, resulting in elimination and prevention. Some of these plasma cells then become memory plasma cells that surveil and prevent re-infection by rapid reactivation upon re-exposure to the antigen. This is to say that conventional vaccines have focused quantitatively on the humoral response [65] as a measure of prevention and protection with acknowledgment that the cellular response mostly contributes to infection control [64]. The cellular response is much better understood today than when antibody titers were first drawn, and it is known that most viral clearance relies heavily on cytotoxic function [66], which has informed the direction of current vaccine development [52,67,68,69,70,71]. The DNA vaccine has shown its ability of inducing strong T cell-mediated immune responses, which is crucial for eliminating infected cells as well as for anti-tumor effects [72]. Interestingly, the delivery method may direct the immune responses for DNA vaccination. For example, the proliferation of CD4 T helper cells toward Th1 or Th2 has been shown to be somewhat directable, with intradermal (ID) delivery suggested to preferentially induce the Th1-immune response by recruiting more APCs and inducing a more polyfunctional cellular immune response, while intramuscular (IM) delivery results in a stronger humoral, or Th2-mediated, immune response with an advantage in antigen expression [65,73,74,75,76]. However, an argument has been made that the delivery method (e.g., gene gun vs. IM depot) is likely to be a more directive factor for the DNA vaccine [75,77]. But, the issue seems to be only supplementary, because adjuvants are also capable of specific stimulatory effects directing CD4+ T cells toward either, or both, Th-1 and Th-2 [78].

Lastly, unlike injected protein or subunit antigens, which have a comparatively short half-life [23,79,80], plasmid DNA can provide tissue expression of antigens over much longer periods of time, potentially offering a better priming of the immune system [39]. Interestingly, in conventional vaccine development, DNA is considered a contaminant since a DNA plasmid is often used to generate a protein or to knock a gene into a bacterium but the plasmid and/or DNA is then intentionally removed in the final vaccine product [81].

## 3. Design

Conventional, non-nucleic acid, vaccine platforms rely on attenuated and/or inactivated pathogens or components of pathogens. The idea of epitope-based vaccine engineering was first put forth in 2014 [82]. Gene vaccines allow the antigen to be designed within the parameters of the intrinsic biochemical machinery; the antigen is customizable within this framework. For most existing pathogens, a target antigen is chosen with an eye towards an antigen that is easily translated and convenient to manufacture, of which the ‘spike’ protein of the SARS-2 coronavirus is an example [83]. However, the antigen selection process can be optimized, and recent advances in both computing power and global collaborative efforts have generated repositories of antigen structure, peptide sequence and nucleic acid sequence, in order to accelerate vaccine development [84,85,86,87,88,89]. The National Institute of Health (NIH) has developed a number of these repositories through the National Center for Biotechnology Information (NCBI) while other repositories are hosted by academic and corporate organizations. Additionally, epitopes can be predicted by simulation, and this offers expedient development for emerging pathogens [84]. 

Immunotherapy is the principle of harnessing the body’s immune system to treat disease, including cancer [90]. Chimeric antigen receptor T (CAR-T) cells [91,92], as an example, have revolutionized the treatment of hematologic malignancy. One of the most exciting applications of DNA vaccines is immunotherapy. Other anti-cancer vaccines exist, but to date these are all preventative cancer vaccines. The widely used vaccines against HPV, a known cause of cervical and oropharyngeal cancers, prevent viral infection, thus preventing HPV-associated cancers [93]. However, the cancer vaccine in the immunotherapy realm more commonly refers to vaccination given against an incident cancer, a therapeutic cancer vaccine [13,43]. 

**Figure 3:**
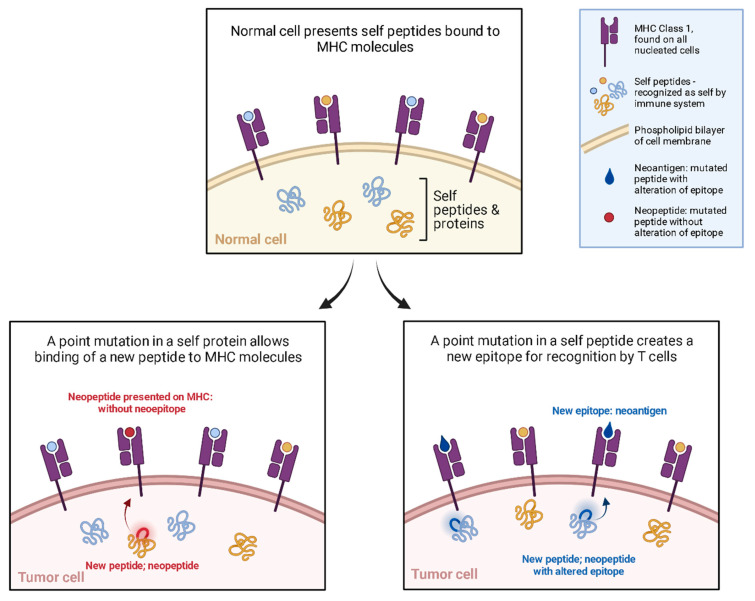
Neoantigen mechanism, a DNA vaccine immunotherapy target. Neopeptides occur when DNA mutation results in a novel transcriptional template leading to a novel polypeptide. Neoantigens occur when the neopeptide is presented on MHC, with the new portion of the polypeptide oriented for exposure to immune cells as a novel epitope. Adapted from “Neoantigen Presentation”, by BioRender.com (2023). Retrieved from https://app.biorender.com/biorender-templates accessed on 10 December 2023.

Immuno-oncology research now includes DNA vaccines. In contrast to the traditional DNA vaccine targeting infectious pathogens, DNA cancer vaccines target cancer cells to induce a generalized as well as a tailored immune response. Cancer cells survive by evading the immune system, this is termed the tolerogenic effect [94]. The most famously shown mechanisms are CTLA-4 and PD-1 [95]. However, cancer cells are constantly forming new peptides by means of genomic mutation, dysregulated RNA splicing, disordered post-translational modification, and integrated viral open reading frames [96]. These new peptides are called neopeptides (Figure 3); some of these neopeptides are ultimately presented on MHC in such an orientation as to present a novel portion of this neopeptide, resulting in a neoantigen. It is these neoantigens that offer a potential target for immunotherapy. The advances in sequencing, and the aforementioned explosion in bioinformatics offer potential personalized anti-tumor immunotherapy targets [97,98].

In the cancer-immunity cycle [99], it is the cytotoxic T cells which provide the greatest anti-tumor effects. While many solid tumors are resectable, metastases and diffuse tumor burdens are often beyond surgical options. While it has been shown that natural killer (NK) cells are more effective for metastatic cancers than cytotoxic T cells are, the addition of intrinsically generated, specific, cytotoxic T cells by immunotherapy offers a promising addition to the repertoire. Many hurdles remain, including waning immunity with age, immunosuppressive treatment regimens and the rapid mutation of tumor cells [100]. 

In cancer vaccine practice, a tumor cell sample is gathered and the somatic mutations from the whole genome or exome are identified. From there, experiments and algorithms help to identify the most suitable ‘neopeptides’, and the appropriate DNA sequences are loaded in to a viral vector or the gene is loaded in to a plasmid to be injected into the patient [101]. This is the framework in which ‘personalized’ DNA vaccines [102] are presented and the idea behind anti-tumor vaccines [96,97,103], many of which are currently in clinical trial [104]. Similarly, known tumor features and markers can be targeted, such as ERBB2 in some breast cancers [40]. 

Existing immunotherapies, like checkpoint inhibition, may also benefit from this additional neoantigen DNA vaccine against cancer cells in much the same way that highly active antiretroviral therapy (HAART) works as a cocktail therapy; to not only reduce the viral load in HIV patients but also to reduce the likelihood of resistance to any individual therapy [105,106,107].

Epigenetic changes to oncogenes, proto-oncogenes, tumor suppressor genes and other related DNA sequences are known causes of cancers [108,109]. Vaccines have been shown to have heterologous, or ‘non-specific’ effects, which are additional effects beyond the specific protection against the targeted disease [110,111,112]. While the exact mechanisms are only beginning to be understood, there is emerging evidence that epigenetic mechanisms are involved [113], including DNA imprinting, as has been shown in HIV and BCG vaccine patient analyses [114,115]. The data from the BCG cohorts indicates that many of these ‘non-specific’ effects can be especially useful, having proven benefit in protecting against autoimmune diseases including multiple sclerosis and type 1 diabetes. Interestingly, the protection conferred accumulates gradually over years [115,116,117]. This is especially interesting in light of recent evidence for the transmission of trained immunity in mammals linked to the epigenetic modification of cell lines, termed, ‘trained immunity’ [118]. 

In principle, epigenetic effects hold promise; however, concerns abound. DNA interaction, especially plasmid integration, is the most feared consequence of DNA vaccination [119]. Although integration has been proven to be exceedingly rare [119,120,121], it is poignant for its catastrophic potential. While DNA integration is feared, activity above the genome may yet prove useful with directed action. This is still an emerging field, and no DNA vaccines to date intentionally target epigenetic changes.

DNA vaccines are, like all DNA, identifiable and functional based on the sequence of nucleic acids. The deployment of the desired antigen necessarily must consider the conveyance apparatus, such as a plasmid or viral vector, as well as diffusion facilitators and adjuvants [122], while also considering the route of administration. The size of the DNA sequence is an important consideration as well. Meanwhile, some small, non-polar molecules (about 40 kDa) likely can diffuse freely across the nuclear envelope [123]. However, for larger molecules (AAV viral proteins are all larger than 60 kDa [124]) the most important mechanism for larger nuclear translocation involves nuclear pore complexes (NPCs) [24,125,126,127]. 

In theory, a DNA vaccine consists of the DNA sequence encoding the potential antigen under the control of a eukaryotic promoter to drive gene expression in the host cell. To date, most DNA vaccines have been bacterial plasmids engineered for gene expression in eukaryotic cells by the selection of appropriate promoter and termination sequences, as well as a nuclear localization signal [128,129]. Plasmids used experimentally, or therapeutically, to deliver gene products are called plasmid vectors. In addition to the gene product, these plasmids also contain an enhancer, a promoter, a transcription termination and/or a polyadenylation signal sequence, and an origin of replication. Additionally, plasmids may contain an antibiotic-selectable marker to enable growth and manipulation in bacterial cells and a marker sequence to identify transfected cells [130]. Engineering plasmids for optimal transfection efficacy and immunogenicity has shown that the promoter is an especially important consideration. Virally derived promoters have been shown to provide greater gene expression than other eukaryotic promoters and, in particular, the CMV promoter, have often been shown to direct the highest level of transgene expression compared with other promoters [131].

Natural elements of plasmid DNA are also important potentiators and modulators of the innate immune response preemptory of the intended, adaptive response. The innate immune system uses pattern-recognition receptors to sense pathogens and then induce the downstream production of interferons and cytokines. One such example is the Toll-like receptor-9 (TLR9), a cytosolic receptor, that binds DNA sequences containing unmethylated cytosine-guanine (CpG) motifs, leading to the activation of signaling pathways [132]. 

Viral vectors were first elucidated in the 1970’s as a way to deliver new genetic material to cells [133]. Naturally, the technology has been deployed in basic science and gene therapy, especially where integration is the goal. When a chosen gene is engineered in to the viral genome, it is considered recombinant. The main classes of viral vectors (including RNA and DNA, single stranded and double-stranded) can be categorized into two groups according to whether their genomes integrate into the host (retroviruses and lentivirus) or persist in the cell nucleus predominantly as extrachromosomal episomes (adeno-associated virus, adenovirus, and herpes viruses) [134,135]. Additionally, each type of vector has characteristics which make it’s use more or less desirable in a given situation, AAV vectors carry a small ssRNA genome, are not very immunogenic but can only accommodate about 5 kilobase (kb) inserts which can integrate in to the genome [136,137,138]. Naked dsDNA adenoviruses possess a larger packaging capacity of about 7.5 kb of foreign DNA, and possess the endogenous virus nuclear localization signals [129]. Importantly these do not integrate and result in short-term episomal expression of the gene of interest in a relatively broad range of host cells, which can be targeted to specific cell populations with structural modifications. The original adenovirus vectors generated strong immune responses, whereas the so-called gutted second and third generation vectors containing deletions have proven to elicit substantially reduced immunogenicity [139], which may be good in gene therapy, but in vaccines the viral capsid immunogenicity is considered advantageous for the early, innate immune priming of the immune system. Previous exposure and immunity to the vector viruses can inhibit vector efficacy. Comprehensive reviews of specific viral vectors exist [134].

The first ZyCov-D, the first DNA vaccine approved for human use, is a plasmid vector [29]. However, both plasmid and viral vector DNA vaccines are currently in pre-clinical or clinical trials [25,140,141]. 

The engineering of the nucleic acid sequence is a source of continued advancement. The improved optimization of the promoter sequence can increase the production of antigens, resulting in a more robust immune response. Likewise, codon optimization, the selection of codon triplets that have the highest tRNA utilization frequency in the cytoplasm, can increase translation rates and mRNA stability [12]. Additionally, DNA vaccines can easily be made multivalent, simply by adding additional antigenic genes to the vector, and this has been shown to enhance their efficacy [12,142]. Likewise, an alternative approach involves co-injecting with adjuvant gene vectors [30].

Differentiating Infected from Vaccinated (DIV) strategies are widely used in animal vaccination campaigns to assort herds in disease control and eradication. Previously called ‘marker vaccines’, these are typically specific protein antigens not present in the natural pathogen, so that an assayable result indicates the animal has been vaccinated as opposed to infected [143,144]. DNA vaccines are perfectly situated for the adoption of ‘markers’, whereby an additional gene product can be used to screen for vaccination, assisting in both the long-term research of vaccination success while also providing readily identifiable vaccination history when uncertain.

## 4. Delivery

Viral vectors often harness the existing pathogenicity, facilitated uptake and existing affinity for permissive cell attachment inherent to the virus [25]. Modifications can be made to the capsid by the attachment or removal of ligand molecules. Because viruses naturally infect cells, most of the molecular engineering concerns plasmid delivery. Optimizing the route of the administration of the vaccine, be it oral, intramuscular or subcutaneous, applies to all formulations.

Interestingly, the seminal DNA vaccine research employed a technology that is still in use today: the biolistic system, also known as the ‘gene gun’ [22]. The earliest DNA vaccines, small plasmids, resulted in relatively low immunogenicity, and this is believed to have been the result of limited plasmid uptake [42]. Several possible reasons have been theorized and substantial work has been done to develop newer delivery methods and to enhance transfection. Physical methods of delivery are thought to be one method to increase the transfection efficiency of DNA vaccines. These methods include particle bombardment and high-pressure delivery, dermal patches (the microneedle array), epidermal tattoo and electroporation (EP). 

The particle bombardment, or ‘gene gun’, approach uses a highly pressurized stream of microscopic heavy metal beads coated with DNA vaccines, similar to the biolistics of the earliest DNA vaccine, to deliver DNA plasmids, linked to microscopic, coated, gold particles [145,146,147] into the skin in a dry powder formulation. High-pressure delivery is conceptually similar to particle bombardment by forcing vaccines in liquid form through a tiny opening to create a fine, high-pressure stream that penetrates the skin [6], without the need for a needle stick [148]. Ultrasound is also being developed for a needleless delivery system through a process called ‘cavitation’, whereby openings are generated in the dermis, through which the vaccine can be delivered without a needle stick [149].

Another concept under investigation, tattoo gun delivery, harnesses the unique properties of the immune system within the epidermal layer. While intradermal vaccination dates to Jenner’s smallpox vaccine [150,151] and is not new, the epidermal idea has increased the deliverable dose by expanding the delivery area to a larger surface by moving across the epidermis [152]. Tattooing creates a dermal and epidermal inflammatory trauma and thus an inflammatory response [152]. Harnessing the natural inflammatory response as the optimal time to deliver plasmids has demonstrated robust immunogenicity [153,154].

At the same time as delivery, electroporation works by delivering short electrical impulses, which are thought to disrupt the plasma membrane of cells to facilitate plasmid uptake [153,155]. Likewise, ‘sonoporation’ uses ultrasound waves to generate openings in the cell membrane [156,157]. 

New research seeks to target specific cells and tissues to protect the nucleic acid vaccine against degradation. Molecularly, lysosomal inhibitors have been shown to protect nucleic acid vaccines [158]. Genetically, this can be achieved by encoding specific proteins, especially fusion proteins, to preferentially engage APCs, thus facilitating the immune response [159,160,161,162]. The mucosal delivery of the DNA vaccine has been hampered by limited vaccine uptake [163,164]; mucosal M cells can be targeted by the conjugation of stimulatory and/or localizing ligands to selected viral vectors, allowing for specific uptake at mucosal surfaces [161]. This method of conjugation can be applied to any conveyance method, so that specific stimulatory molecules can be attached to liposomes or to nanoparticles as well. In a similar way, gene adjuvants, discussed later, are another convenient mechanism for stimulating local immune cells. 

**Figure 4:**
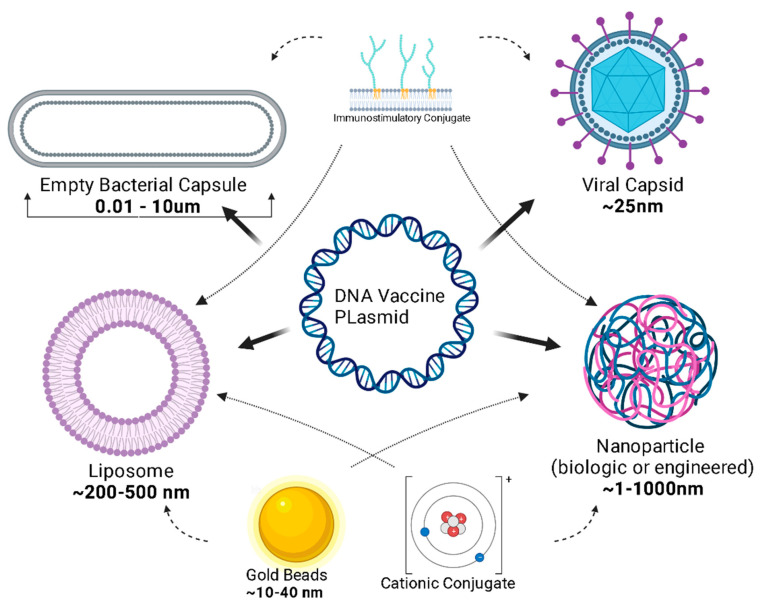
DNA Vaccine transport across the plasma membrane. For DNA vaccines to have the intended effect, the DNA must be transported across the plasma membrane and the DNA must then enter the nucleus. Nuclear localization signals are often intrinsic to DNA viruses but can be engineered in to plasmids. Plasmid transport across the plasma membrane can be facilitated in many ways, some of which are represented here. Clockwise from viral capsid. Viral capsid (most often as viral vector) protects and facilitates delivery of nucleic acid cargo to cytosol of cells by harnessing viral properties for cellular engagement and cargo routing. Nanoparticles can be engineered to small sizes with lipophilicity and non-polarity to facilitate transportation of plasmid across the plasma membrane. Cationic conjugates can be attached to negatively charged nucleic acids of plasmids to facilitate transport across plasma membrane. Coated gold beads are used as carrier molecules similar to engineered nanoparticles and can easily cross the phospholipid membrane to reach the cytosol. Liposomal covering allows for passive diffusion across lipid bilayers including the nuclear envelope. Empty bacterial capsules contain intrinsic immunostimulatory properties and can prevent extracellular breakdown of naked nucleic acid. Created with BioRender.com.

Plasmids must cross the phospholipid cellular membrane, avoid breakdown by cytosolic organelles and nucleases, and translocate across the nuclear envelope. Nanoparticles and viral vectors harness existing technology to optimize the delivery of the vaccine to its site of action, the nucleus [165]. Liposomes are a popular delivery mechanism because of their diffusion across the phospholipid membrane; liposomes also have adjuvant properties [165]. Like liposomes, engineered nanoparticles can be designed for lipophilicity and can be constructed to micrometer diameter sizes (Figure 4). 

Nucleic acids carry a negative charge and DNA vaccine plasmids carry a net negative charge, and since most membranes of living cells are considered to have a resting negative charge, similar in principle to the idea of electroporation [153], it is likely that the attenuation of the charge or the masking of the charge will facilitate a better uptake of the plasmid [12,166]. Polycations have been trialed; however, encapsulation has proven, thus far, to be a better solution [12].

Before plasmid uptake, engineering the vaccine vector is a field of exciting growth. Previously, naked plasmids needed to be deposited in relatively large concentrations. Novel ideas about the delivery vector have expanded the scope and efficacy of DNA vaccines [167]. The typical DNA plasmid can be attached to a nanoparticle (<100 nm), which is a microscopic synthetic polymer such as starch, cellulose, silk, collagen, gelatin, albumin and chitosan (from chitin) chosen for biocompatibility, biodegradability, and low toxicity. In recent years, several studies have focused on the advances in this field [168]. Alternatively, cytoplasm-free, non-denatured Gram-negative bacterial cell envelopes, known as “bacterial ghosts’, are being explored for their inherent cell and tissue affinity, ease of production, and storage capabilities that do not require refrigeration, as well as their potential to inherently induce the innate immune system [39,167]. 

And, as mentioned before, the site of DNA vaccine delivery has been shown to affect the immune response and thus the efficacy of the vaccine [169]. 

Moreover, DNA vaccines are known to be very stable at room temperature, which is of significance for both transport and storage [55,170]. 

## 5. Adjuvants

**Figure 5:**
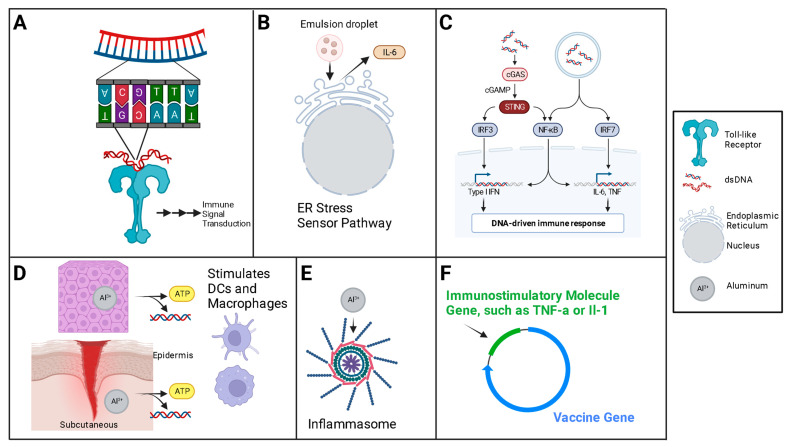
Common adjuvants and proposed mechanisms. (**A**) The CpG motifs stimulate TLRs, which activate signal transduction cascades, leading to nuclear upregulation of cytokines which stimulate immune cell response; most famously, the STING pathway. (**B**) Oil emulsions stimulate endoplasmic reticulum (ER) stress signaling pathway, which upregulates inflammatory cytokines, like interleukin-6 (IL-6), which activate and potentiate immune cells. (**C**) DNA stimulates signal transduction cascade, which upregulates transcription of inflammatory genes. (**D**) Aluminum causes inflammation and stimulates a release of ATP and DNA from injured cells, which activate DCs and macrophages. (**E**) Aluminum also stimulates inflammasomes. (**F**) Gene Adjuvants are immunostimulatory genes added to the plasmid for transcription within the cell (or can be delivered as separate plasmids alongside the vaccine), which are transcribed with the antigenic gene and work to potentiate immune cell response. Created with BioRender.com.

DNA is itself an adjuvant when exposed in the cytosol (Figure 5). The interplay of the many recognition molecules converges on a major molecule in the relay pathway to the innate immune response, the STING, or the stimulator of the interferon gene [171]. The two major classes of ‘exogenous’ adjuvants used for DNA vaccines are the traditional, chemical [10] adjuvants, and, second, the gene-encoded adjuvants—proteins encoded by the DNA vaccine or in plasmids delivered with the DNA vaccine. 

Traditional adjuvants, such as aluminum salts and adjuvant systems, which are commonly used in conventional vaccines, activate the local innate immune system at the site of delivery and/or delay the removal of the vaccine from the site of delivery. Nucleic acid vaccine plasmids can be modified to include gene products in addition to the vaccine antigen. Genetic adjuvants, or gene adjuvants, are most commonly cytokine genes placed in the plasmid, or a separate plasmid, to be transcribed with the antigen [165,172,173,174]; other gene products are possible, with Toll-like receptor (TLR) ligands a common choice as well [175]. 

Plasmid DNA has historically been derived from bacteria. Because of its fundamental design, DNA plasmids have a number of CpG motifs. These are often unmethylated in non-mammals [39], and it is specifically unmethylated CpG which is immunostimulatory [176,177], capable of stimulating innate immune responses via the TLRs [178]. Specifically, the unmethylated CpG motif represents a pathogen-associated molecular pattern (PAMP), which is recognized by a specific pattern recognition system in the TLR-9 of antigen presenting cells [60,179]. Interestingly, TLR activation has been shown to have anti-tumor effects, and this synergy has inspired much of the cancer vaccine research [180]. 

Co-delivery with cytokines and/or cytokine genes, especially interleukin (IL) -2, -12 and -15 has been shown to augment the adaptive immune response by stimulating APCs [165,181]. Likewise, immunopotentiators, like TLR ligands, have been shown to similarly enhance the initial and long-term immune response after DNA vaccine delivery [132,182,183]. Traditional adjuvants, like aluminum-based compounds, still have a place in the DNA vaccine world, as do co-delivered CpG nucleic acid motifs [184,185,186], which are ironically mostly methylated in mammalian genomes (silencing genes) [187], but can be engineered into DNA plasmids unmethylated [39,188,189].

## 6. Limitations/Concerns

The great fear with DNA vaccines is genome integration; this could be a partial or complete plasmid genome integration, which can result in insertional mutagenesis, possibly in the activation of oncogenes or the inactivation of tumor suppressor genes [190]. The FDA designed guidelines for DNA vaccines, stating that the frequency of plasmid integration needs to be lower than the spontaneous mutation rate [81]. All of the data to date suggest that while integration can occur, it occurs at a frequency below the rate of spontaneous mutagenesis [120,121,191]. DNA plasmids can remain at the site of injection for months after injection, typically longer than RNA can remain, due to the protection afforded by double stranding [41]. DNA poses a greater risk than RNA for genome integration because RNA requires a reverse transcriptase to generate cDNA prior to potential integration. While most cells have a reverse transcriptase, it is not frequently expressed at very high levels [192,193]. The potential integration of the DNA vaccine remains a concern in the public consciousness [194,195,196], and we cannot discredit the stochastic evidence since the integration rate is additive to the spontaneous rate of mutation, which is, per gene, on the order of one mutation per one million cell divisions in humans [197,198]. Necessarily, a harmful mutation must be even less frequent. Nevertheless, published data overwhelmingly support the safety of DNA vaccination, the convincing example being the approval of the first DNA vaccine, ZyCovD [28]. Nonetheless, public concern is a serious impediment to gene vaccine uptake, as evidenced during the COVID pandemic [199,200,201]. 

Many approved vaccines including the Tetanus, Diptheria and acellular Pertussis (TDaP), and the Measles, Mumps, Rubella and Varicella (MMRV), have much higher (although still low) serious adverse event rates [202]. Public awareness campaigns as well as long-term COVID DNA vaccine safety experience and data will likely exonerate DNA vaccines in this regard, although this will need to be re-demonstrated with many of the newer technological advancements as alterations of the plasmid DNA, adjuvants and alternative delivery vehicles could theoretically affect the probability of integration. 

Second, DNA plasmids are often developed in bacterial lines, and these lines are often selected for by inserting an antibiotic resistance gene into the plasmid. This has led to a concern about antibiotic resistance as a possible adverse effect of DNA vaccination should the antibiotic resistance gene be integrated [203]. This has not been shown in any trials to date and has been proven unlikely [204]. Given the very low rate of insertion and the absence of single gene transfection to date [205,206,207], this issue will benefit from concerted educational campaigns and repeated evidence. Repeat data may be harder to generate because the FDA has proposed that DNA vaccines prepared using a plasmid DNA previously documented to have an acceptable DNA biodistribution/integration profile could waive biodistribution/persistence studies. Additionally, newer bacterial-plasmid systems without antibiotic resistance genes are under investigation [208]. One bacterial-free DNA amplification system, the doggybone, has demonstrated an equivalent immune response from an entirely enzymatically constructed linear DNA vaccine [209].

Another concern is possible antigen tolerance after continuous exposure to DNA vaccine products. There is evidence that peripheral immunity can develop, thus having the opposite effect to vaccination [210]. This is very rare but still possible. As noted previously, nucleic acids are available for transcription until degradation, and while DNA vaccine plasmids are designed to be non-replicating [30], plasmids can be transcribed multiple times and can remain at the site for months after delivery [41]. This has purposeful application in the prevention and treatment of autoimmune disorders by intended, tolerizing DNA vaccines [211,212,213,214]. However, after DNA vaccines deliver nucleic acid to cells, the lifespan of the DNA vaccine is finite but dependent on numerous factors [215]. Repeated exposure to antigens has the potential to result in immunological tolerance [210,216,217]; the failure to recognize an antigen as a non-host due to prolonged exposure, in which case the immune system does not mount a response [192,198,199]. Tolerance could be problematic not only at the individual level but at the population level as well, because those vaccinated could ultimately become permanent carriers [218]. It might reasonably be assumed that injected plasmids could also induce anti-DNA antibodies, but this has been proven untrue to date in published studies [219]. Further investigation needs to be conducted to determine the impact of the prolonged expression of antigens by DNA vaccines on host responses.

## 7. Conclusions

**Figure 6:**
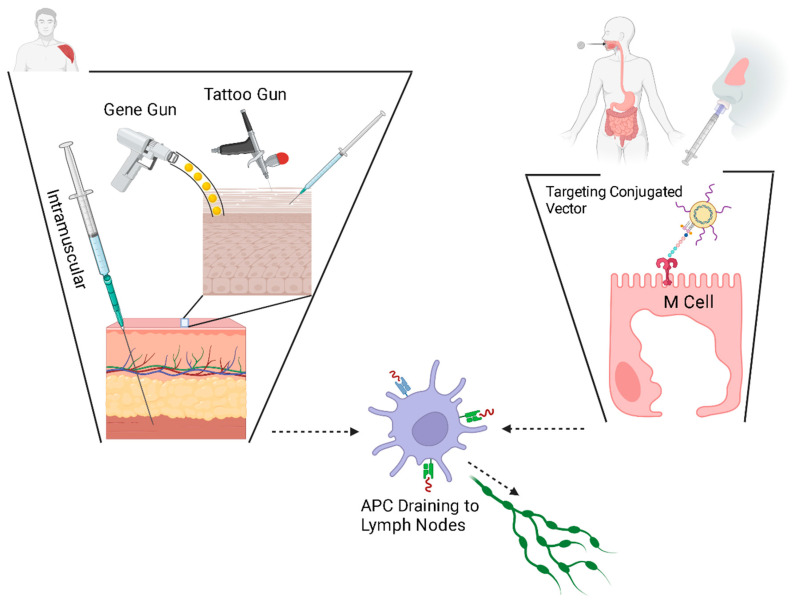
Mechanism of vaccine delivery and intended site of vaccine delivery. All vaccines ultimately stimulate antigen presenting cells (APCs) which drain to lymph nodes to further activate the adaptive immune response. Epidermal, intradermal and intramuscular (IM) delivery routes rely on different, but related, populations of macrophages for adaptive immune system activation (**on the left**). Mucosal delivery is enhanced with specific M-cell stimulatory molecules (**on the right**). Created with BioRender.com.

DNA vaccine approval was accelerated by a worldwide need for a scalable and stable vaccine against SARS-2-CoV in 2020 [27,220]. However, DNA vaccine technology has been rapidly improving and advancing since first proposed in the early 1990s. Plasmid engineering has advanced, with robust promoters, immunostimulatory CpG motifs and immunostimulatory gene adjuvants now part of the design. BLAST sequencing and other bioinformatic tools [221,222] allow for rapid selection of robustly immunogenic antigens, and plasmids can be easily made polyvalent. Viral vectors are widely used in many DNA vaccines in development, hindered mainly by their limited load size and immunogenicity. Plasmid vectors can be transported in empty Gram-negative bacterial cell envelopes for their immunostimulatory effects, or plasmids can be attached to liposomes or nanoparticles to facilitate diffusion across the plasma membrane. The biolistic method, whereby DNA is forced into the cell, from the first successful study of DNA vaccines, remains under investigation. Likewise, the plasma membrane can be disrupted by sound and current. Some vectors now include cell-population-targeting motifs, most specifically to facilitate mucous membrane delivery. The immune micro-environment of different tissues is now better understood than ever before, with tattooed delivery potentially yielding immune response advantages in epidermal APC populations (Figure 6). These technologies can be combined in seemingly infinite ways. For instance, polycations are attached to liposomes to engage both the hydrophobic external plasma membrane as well as the typically negative membrane potential of most cells. 

DNA vaccine technology is an emerging tool in the fight against cancer in the form of immunotherapy. Personalized medicine is a buzzword. Neoantigens may be appropriate targets for stimulation of the immune system against tumors and leukemias, and DNA vaccines can be assembled specifically and rapidly [223]. Likewise, epigenetic changes may be able to impart protective factors, both for the pathogen of interest and for future disease. Exciting evidence suggests that induced epigenetic changes may even be heritable, suggesting the possibility of lineage vaccination.

DNA vaccines hold demonstrated promise for global vaccination campaigns. They are stable, compact, scalable and reasonably simple to administer, depending on the delivery method. The discovery and development of optimal antigens is the key challenge; many exciting scientific and engineering advances occur regularly, beckoned by enormous potential. The great fear of genome integration is seemingly over-weighted, it being incredibly rare. Just as with all other vaccines approved for the general population, the continued adherence to, and updating of the FDA regulations [224,225] for monitoring adverse outcomes will lead to step-wise improvements in the safety of DNA vaccines. Compared to conventional peptide or subunit vaccines, the common side effect profile is somewhat more severe. However, the COVID vaccines provide the only gene vaccines for comparison to date [226], and it is likely that the specific antigen contributes more substantially to the varying side effects of different vaccines than do the vaccine technologies themselves. Conversely, at the population level, the serious adverse event rate is much lower for gene vaccines compared to most conventional vaccines [202]. However, at the individual level, the potential side effects of DNA vaccines are potentially more catastrophic, and this is a relatively unique paradox; a lower risk of a serious event but a higher risk of a catastrophic event. The advantages of DNA vaccines are clear, and the trajectory is one of rapid ascent. Careful policies, including ethical considerations and clearly defined measurables regarding both persons and populations, are essential to avoid anything like the decades-long delay suffered by gene therapy in the early 2000s [227,228,229]. 

## Data Availability

Not applicable.
